# Polyglycolic Acid Polymer Versus Porcine Small Intestine Submucosa Graft After Plaque Incision in Peyronie's Disease: A Comparative Study

**DOI:** 10.1111/andr.70151

**Published:** 2025-11-24

**Authors:** Renan Trevisan Jost, Leonardo Seligra Lopes, Pedro Caetano Edler Zandoná, Jovânio Fernandes da Rosa, Pedro Fernandes Lessa, Felipe Placco Araujo Glina, Nívio Pascoal Teixeira, Sidney Glina

**Affiliations:** ^1^ Hospital Governador Celso Ramos Florianópolis Santa Catarina Brazil; ^2^ Disciplina de Urologia Centro Universitário FMABC Santo André São Paulo Brazil

**Keywords:** erectile dysfunction, graft, penile curvature, penile deformity, peyronie's disease

## Abstract

**Background:**

Peyronie's disease (PD) with severe penile curvature often requires surgical correction through plaque incision and grafting (PIG).

**Objectives:**

To compare surgical outcomes between polyglycolic acid polymer grafts and porcine small intestinal submucosa (SIS) grafts in patients undergoing PIG for PD.

**Materials and Methods:**

This retrospective cohort study included patients with PD who underwent PIG using either Gore Bio‐A (BioA group, *n* = 20) or SIS grafts (Cook Biotech, West Lafayette, IN, USA; SIS group, *n* = 21). The primary outcome was the incidence of postoperative erectile dysfunction (ED). Secondary outcomes included the degree of penile curvature correction, sensory disturbances, patient satisfaction, and surgical complications.

**Results:**

The median age was 61 years in the BioA group and 57 years in the SIS group (*p* = 0.381). The BioA group exhibited a significantly higher rate of refractory ED compared to the SIS group (*p* = 0.006). No significant differences were observed between groups in terms of penile straightening, sensory alterations, postoperative complications, or the need for penile prosthesis implantation. Patient satisfaction was significantly higher in the SIS group (*p* = 0.015).

**Discussion:**

This study is the first to directly compare Gore Bio‐A and SIS grafts in PIG surgery for PD. Despite similar safety profiles and high curvature correction rates in both groups, erectile function outcomes were significantly better in the SIS group. Patient‐reported satisfaction also favored SIS. Although associated with higher rates of ED, Gore Bio‐A may still represent a viable option in selected cases, particularly when PIG is performed in conjunction with penile prosthesis implantation. Limitations include the retrospective, non‐randomized design, and small sample size.

**Conclusion:**

In this cohort, the SIS graft was associated with better postoperative outcomes than the BioA graft, with lower rates of refractory ED and higher patient satisfaction following PIG for PD.

## 1 Introduction

Peyronie's disease (PD) is a fibrotic disorder of the penile tunica albuginea that can cause penile deformity and pain, which can negatively impact a patient's quality of life. PD poses a significant challenge for sexual medicine specialists because of its complex and incompletely understood nature [1, 2].

The chronic phase of PD is characterized by the stabilization of penile curvature, cessation of the pain, and possibly the formation of a well‐defined plaque on the corpora cavernosa due to fibrosis. In this phase, patients can be offered collagenase injections and/or surgery depending on the curvature degree or deformity complexity [3, 4, 5, 6].

There are numerous surgical techniques for PD deformities treatment such as plication, plaque incision and grafts, and penile prosthesis implant [4, 6]. Shortening procedures are linked to penile shortening and are not recommended for complex cases such as notching, hour‐glass deformity, or ossified plaque. Lengthening procedures, such as plaque incision and grafting (PIG), are suitable for addressing complex curvatures without erectile dysfunction (ED) and are a more appropriate method for multiplanar curvatures. Penile prosthesis implantation (IPP), with or without additional procedures, is the gold standard for patients with ED and PD [7].

The wide variety of available grafts for PD can be overwhelming for surgeons, but most studies were single‐arm studies and had a high risk of bias [8]. There are many types of grafts available, including autologous grafts (venous grafts, such as a penile dorsal vein or saphenous vein, buccal mucosa grafts, dermal grafts, tunica albuginea grafts, and tunica vaginalis grafts), allografts (cadaveric fascia lata and human pericardium, such as Tutoplast from Coloplast, Minneapolis, MN, USA), synthetic grafts (Gore‐Tex from Gore & Associates Inc., Newark, DE, USA; Dacron from Invista, Gloucester, UK and Advansa, Hamm, Germany; and collagen fleece hemostatic patches, such as TachoSil from Corza Medical, Westwood, MA, USA), or xenografts (small intestinal submucosa [SIS], such as Surgisis from Cook Medical, Bloomington, IN, USA, and bovine pericardium) [4, 9, 10, 11, 12]. Autologous grafts (53.5%), allografts (22.7%), and xenografts (15.7%) were the most frequently reported materials [13].

In a recent meta‐analysis, TachoSil graft showed the best performance when preoperative curvature is taken into account, and buccal mucosa grafts were found to result in the highest penile straightening rates and were associated with the least de novo ED [8]. Although many grafts have been proposed to cover the albuginea defect, no strong definitive evidence supports the superiority of one specific graft over others [11, 12].

The SIS graft has been extensively validated in the literature for its safety and efficacy in penile reconstructive surgery [14] and currently represents one of the more commonly used grafts in PD surgery [11, 15, 16]. In contrast, the polyglycolic acid polymer graft is a more recent innovation, with limited clinical data supporting its use [10]. Zandoná et al. [10] reported outcomes following PIG with the polyglycolic acid polymer, demonstrating rates of penile curvature correction and sensory alteration comparable to those previously documented. No major surgical complications were observed. However, a higher incidence of refractory ED was noted, potentially influenced by the inclusion of a cohort with a greater baseline prevalence of ED. These findings warrant further investigation with larger, controlled studies to confirm the safety and functional outcomes of this graft material.

Therefore, the aim of this study was to compare the clinical outcomes of polyglycolic acid polymer graft with porcine SIS in patients undergoing PIG for PD.

## 2 Methods

A retrospective cohort study was conducted involving patients who underwent PIG for PD between 2018 and 2024. Data were collected through a retrospective review of medical records and, when necessary, through follow‐up telephone interviews. Patient confidentiality was strictly maintained throughout the data collection process. Inclusion criteria consisted of patients with stable PD presenting severe or complex penile deformities who were treated with grafting procedures within the study period. Exclusion criteria included the presence of severe ED, documented coronary artery disease, uncontrolled diabetes mellitus, or concomitant insertion of a penile prosthesis at the time of surgery.

Patients were allocated into two groups based on graft type: the BioA group (*n* = 20) and the SIS group (*n* = 21). Randomization was not feasible due to inconsistent availability of the graft materials; therefore, allocation was determined by graft availability at the time of surgery. The grafts utilized included Gore Bio‐A (W.L. Gore & Associates Inc., Flagstaff, Arizona, USA), composed of a bioabsorbable synthetic polymer matrix (67% polyglycolic acid and 33% trimethylene carbonate), approved by the Brazilian Health Regulatory Agency (ANVISA) for use in soft tissue reconstruction, and porcine SIS (Cook Biotech, West Lafayette, IN, USA).

The surgical technique was standardized across all cases. Under general anesthesia, a subcoronal circumferential incision was made, followed by penile degloving. An artificial erection was induced to identify the direction and severity of the curvature. In cases of dorsal curvature, the neurovascular bundle was meticulously dissected from the corpora cavernosa; for ventral curvatures, the urethra was mobilized. A second artificial erection was then performed to precisely identify the point of maximal curvature, at which a double‐Y relaxing incision was made in the tunica albuginea.

**FIGURE 1:**
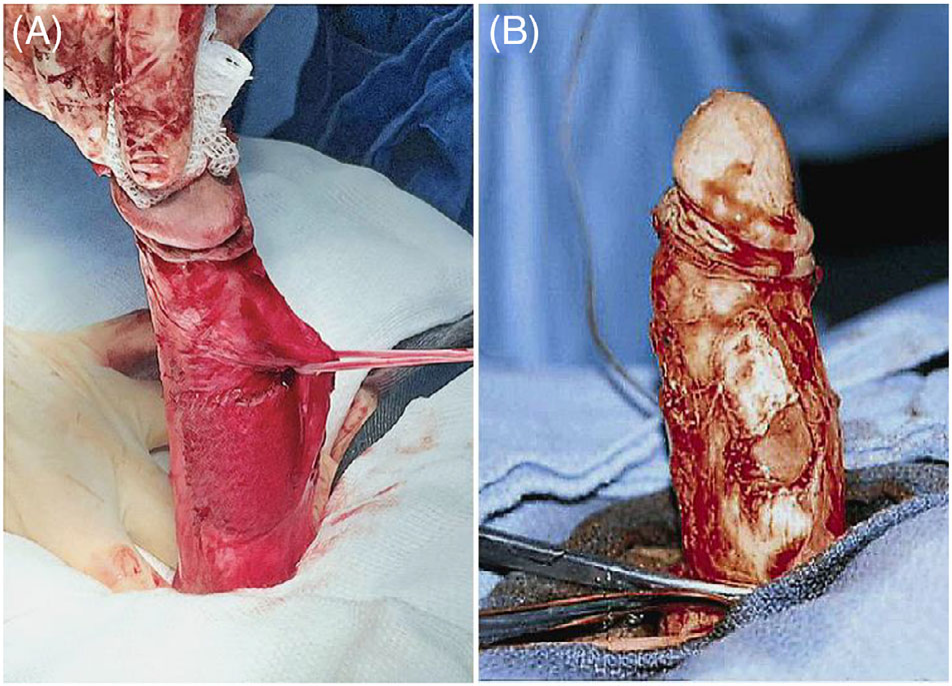
(A) Polyglycolic acid polymer (BioA) graft after plaque incision. (B) Porcine small intestinal submucosa (SIS) graft after plaque incision.

The resulting defect was measured, and a graft—oversized by 30% to account for tissue remodeling—was tailored and secured over the defect using continuous 3‐0 Vicryl sutures along its margins (Figure 1). Hemostasis was achieved by bilateral closure of the Buck's fascia. Penile skin closure and circumcision were performed routinely. A urinary catheter and compressive dressing were left in place for 24 h; suction drains were not used. All patients were discharged within 24 h postoperatively and prescribed tadalafil 5 mg once daily. Resumption of sexual activity was encouraged after 6 weeks.

**FIGURE 2:**
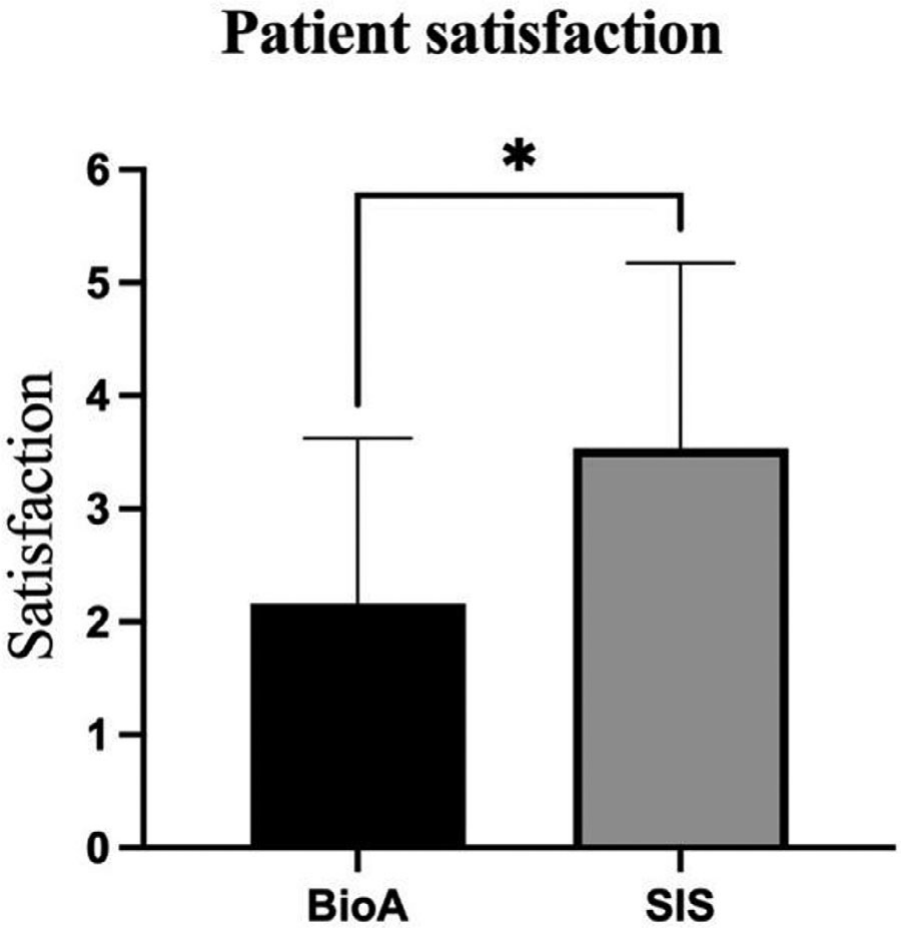
Satisfaction in patients with Peyronie's disease after plaque incision and graft. ^*^ *p* = 0.015, ^*^Mann–Whitney test, significant *p* < 0.05.

All patients underwent clinical interviews and physical examinations during routine postoperative follow‐up visits. In cases where residual curvature was suspected based on patient self‐report, artificial erection was induced in the office to objectively confirm the finding.

The primary endpoint of the study was ED, assessed using the Erection Hardness Score (EHS) [17]. Secondary endpoints included the degree of penile curvature correction—defined as the absence of residual curvature greater than 20°—alterations in penile sensation, patient satisfaction, and postoperative complications.

Refractory ED, whether present preoperatively or developed postoperatively, was defined as the persistent inability to achieve or maintain an erection adequate for sexual intercourse despite the use of phosphodiesterase type 5 inhibitors (PDE5i). All patients were preoperatively counseled regarding the potential risk of worsening erectile function and were informed about the possibility of penile prosthesis implantation as a second‐stage procedure, if necessary. Patient satisfaction was assessed with a five‐point Likert scale [18]. Nevertheless, all patients were evaluated for at least 6 months.

Statistical analysis was performed using GraphPad Prism version 10 (GraphPad Software, San Diego, CA, USA). A *p* value < 0.05 was considered statistically significant. The Shapiro–Wilk test was used to assess the normality of data distribution. As the data did not meet assumptions for normality, non‐parametric tests were employed. The Mann–Whitney test was used for continuous variables, and Fisher's exact test was applied for categorical variables to compare groups and evaluate differences in postoperative outcomes.

The study protocol was reviewed and approved by the Institutional Review Board of the participating hospital (protocol number 47537021.0.0000.5360). Written informed consent was obtained from all participants at the time of enrollment. Psychological counseling was offered to all patients prior to surgery; however, participation in counseling was voluntary and not mandatory as part of the surgical protocol.

## 3 Results

Patient characteristics are summarized in Table 1. No statistically significant differences were observed between groups with respect to age, weight, height, body mass index (BMI), or degree of penile curvature. Table 2 presents the preoperative characteristics of penile curvature in patients diagnosed with PD.

**TABLE 1:** Descriptive characteristics of patients with Peyronie's disease.

	BioA (*n* = 20)	SIS (*n* = 21)	*p*
Age (year), median (IQR)	61 (54–64)	57 (50–64)	0.381^a^
Weight (kg), median (IQR)	69 (60–82)	78 (68–81)	0.260^a^
Height (cm), median (IQR)	170 (166–175)	171 (169–175)	0.479^a^
BMI, median (IQR)	24.62 (21–27)	26.06 (24–28)	0.193^a^
Penile curvature degree, median (IQR)	60 (57–80)	67 (58–90)	0.104^a^
Patients comorbidities			
Hypertension (*n*)	8	9	0.999^b^
Diabetes (*n*)	6	4	0.484^b^
Smokers (*n*)	4	1	0.343^b^
Cardiovascular disease (*n*)	1	2	0.999^b^
Dupuytren's contracture (*n*)	1	0	0.487^b^

^a^ Mann–Whitney test, significant *p* < 0.05.

^b^ Fisher's exact test, significant *p* < 0.05.

**TABLE 2:** Characteristics of penile curvature of patients with Peyronie's disease.

	BioA (*n* = 20)	SIS (*n* = 21)	*p*
Penile curvature, *n* (%)			
Dorsal	10 (50)	10 (47.6)	0.509^a^
Dorsolateral	7 (35)	4 (19)	
Ventrolateral	1 (5)	0	
Lateral	1 (5)	4 (19)	
Ventral	0	1 (4.7)	
Complex	1 (5)	2 (9.5)	

^a^ Fisher's exact test, significant *p* < 0.05.

Table 3 shows preoperative and postoperative functional outcomes. There was a higher rate of refractory ED in the BioA group compared to the SIS group postoperatively (*p* = 0.006).

**TABLE 3:** Preoperative and postoperative functional outcomes of patients with plaque incision and graft for Peyronie's disease.

	BioA (*n* =20) *n* (%)	SIS (*n* = 21) *n* (%)	*p* ^a^
Preoperative erectile function			0.353
EHS 4	17 (85)	17 (80)	
EHS 3	3 (15)	4 (19)	
EHS 2	0 (0)	0	
EHS 1	0	0	
EHS 0	0	0	
Postoperative erectile function			0.006
EHS 4	3 (13)	11 (52)	
EHS 3	2 (9)	4 (14)	
EHS 2	4 (18)	2 (9.5)	
EHS 1	4 (22)	1 (4.7)	
EHS 0	7 (36)	3 (14)	

*Note*: EHS 0: Penis does not enlarge; EHS 1: Penis is larger but not hard; EHS 2: Penis is hard but not hard enough for penetration; EHS 3: Penis is hard enough for penetration but not completely hard; EHS 4: Penis is completely hard and fully rigid.

^a^ Mann–Whitney test, significant *p* < 0.05.

Additional postoperative outcomes following PIG with either BioA or SIS grafts are presented in Table 4. There were no statistically significant differences between groups in terms of penile straightening, sensory changes, or the need for penile prosthesis implantation. Postoperative complications occurred exclusively in the BioA group, affecting two patients: one developed a penile hematoma, and the other experienced complete graft resorption within a few weeks postoperatively. This resorption resulted in a prominent penile bulge during erection, like an aneurysm, with the appearance that no graft had been placed.

**TABLE 4:** Postoperative secondary outcomes in patients with plaque incision and graft for Peyronie's disease.

	BioA *n* (%)	SIS *n* (%)	*p*
Remaining curvature	2 (9)	4 (19)	0.665^a^
Sensitivity changed	2 (9)	2 (9.5)	0.999^a^
Penile prosthesis implantation	6 (27)	2 (9.5)	0.132^a^
Patients satisfaction			0.015^b^
Very unsatisfied	10 (52.6)	3 (18.7)	
Unsatisfied	2 (10.5)	1 (6.25)	
Neutral	3 (15.7)	1 (6.25)	
Satisfied	2 (10.5)	4 (25)	
Very satisfied	2 (10.5)	6 (37.5)	

^a^ Fisher's exact test, significant *p* < 0.05.

^b^ Mann–Whitney test, significant *p* < 0.05.

Patient‐reported postoperative satisfaction is illustrated in Figure 2. Satisfaction was significantly higher in the SIS group compared to the BioA group (*p* = 0.015). In the SIS group, 62.5% of patients reported being satisfied or very satisfied, whereas only 21% of patients in the BioA group reported similar satisfaction levels.

## 4 Discussion

To the best of our knowledge, this is the first study to directly compare Gore Bio‐A and SIS grafts in the surgical treatment of PD, providing novel insights into their clinical performance. Despite decades of investigation, an ideal graft material for tunical defect reconstruction in PD has yet to be established. One of the principal advantages of allografts and xenografts over autologous or synthetic grafts is the avoidance of donor site morbidity, as these materials do not require harvesting from the patient. In addition, they offer the potential for biological integration into the local tissue of the corpora cavernosa, which may enhance healing and reduce the risk of graft‐related complications [19]. On the other hand, a buccal mucosa graft may offer better results in recovery of sexual function than other tissues because it is thick, elastic, and flexible enough to adapt to the edges of the tunica albuginea incision, creating perfect contact between the surfaces. The early integration and good sealing reduce the risk of postoperative ED (3.5%), and patient and partner satisfaction were 85% and 90%, respectively.

In our study, patients who received SIS grafts demonstrated superior postoperative erectile function outcomes compared to those in the BioA group. Similarly, Rosenhammer et al. [19] reported slightly better erectile function outcomes in patients who received SIS grafts compared to those treated with collagen fleece grafts. Valente et al. [20] observed that 17.8% of patients experienced moderate to severe ED following PIG using SIS grafts. Consistent with these findings, 18.7% of patients in our SIS group reported an EHS of 0 or 1, indicative of moderate to severe ED. In contrast, 58% of patients in the BioA group had EHS scores of 0 or 1, suggesting a significantly higher incidence of postoperative ED. This agrees with other findings [10] reporting that 64.2% of patients receiving BioA grafts experienced substantial ED. Preoperative patient selection and counseling are critical for optimizing postoperative erectile outcomes. Patients with adequate baseline erectile rigidity appear to be the best candidates for grafting procedures, irrespective of graft material [19]. Nevertheless, even among carefully selected patients with satisfactory preoperative erectile function, the BioA graft was clearly inferior to SIS in preserving erectile function. Due to its rigidity, the BioA material may not adequately adapt to the surface of the tunica albuginea; the lack of plasticity favors blood leakage through the suture edges can explain more ED in these patients. SIS, being more malleable and elastic, may seal the suture edges better.

The remaining curvature was lower in the BioA group than in the SIS group, but not statistically significant. According to the EAU guideline [21], the curvature correction was success for 83.9% (range 54–91) using SIS, 87.4% for bovine pericardium (range 76.5–100), and 81.2% for dermis (range 60–100). This result was similar in our study: the BioA group presented 91.4% curvature correction and SIS group 81.9%.

Regarding decreased glans sensitivity, we obtained 8.6% in the BioA group and 13.6% in the SIS group. Similarly, Zandoná et al. [10] found that only 7.1% of the patients had a decreased in sensitivity using BioA graft. Chung et al. [9] reported 13% of impaired sensitivity after dermal graft, and Horstmann et al. [22] found 16% after using TachoSil, but slightly higher compared to the series by Sansalone et al. [23] with 3%, after bovine pericardium.

Patient‐reported satisfaction was notably higher in the SIS group, with 62.5% of individuals reporting being satisfied or very satisfied postoperatively, compared to only 21% in the BioA group. This discrepancy is likely attributable to the higher incidence of postoperative ED and the greater number of patients in the BioA group who ultimately required penile prosthesis implantation, both of which may have negatively impacted overall satisfaction. Using SIS graft for Peyronie's surgery, Valente et al. [20] showed a greater overall satisfaction regarding sexual activity after surgery in 65.7% of patients.

The absence of major surgical complications, especially graft rejection, infection, or extrusion, seems to be a characteristic of bioabsorbable materials. In the BioA group, one patient developed a hematoma, and another presented with ED and a bulging appearance at the graft site. The latter required reoperation within the first few postoperative weeks. Intraoperatively, the graft appeared to have undergone complete resorption, as though no material had been placed. A new graft was placed using an SIS patch, with a favorable postoperative outcome. The complications observed in patients who received the synthetic BioA graft may be attributable to the material's rigidity, which could promote blood leakage along the suture line and result in hematoma formation. In contrast, SIS, being more pliable and elastic, may provide a better seal at the suture interface, minimizing this risk.

Although two patients in the BioA group experienced complications, we believe these events did not compromise the overall safety of the material. Considering the absence of major surgical complications, we infer that the BioA graft demonstrated a safety profile comparable to that of SIS in PIG procedures for PD.

Although we do not have precise operative times, we do not believe that the BioA graft increases operative time, as the technique is identical to that used with SIS, and we encountered no technical difficulties attributable to the graft's greater rigidity. In our view, the BioA graft is technically feasible, with no placement difficulties and no serious complications. Even if associated with higher rates of ED, the BioA graft may still represent a viable option in selected cases, particularly when PIG is performed in conjunction with penile prosthesis implantation, given its favorable safety profile and low complication rates.

Our study has several limitations. This retrospective, non‐randomized, single‐center cohort has a relatively small sample, which limits statistical power, especially for infrequent events. Allocation by graft availability (rather than randomization) introduces potential selection bias and unmeasured confounding. Follow‐up intervals were heterogeneous and not uniformly documented, potentially affecting the ascertainment of medium‐ to long‐term outcomes. Data were derived from chart reviews and telephone interviews, making outcomes susceptible to recall and reporting bias; assessors were not blinded. Objective physiologic testing (e.g., penile duplex Doppler) and standardized penile length measurements (pre‐ and postoperatively) were not routinely performed. Intraoperative variables, such as exact defect size, graft area, suture‐line length, and operative time, were not systematically recorded. Although baseline characteristics did not differ significantly between groups, the study was underpowered to exclude clinically relevant imbalances; consequently, no multivariable adjustment or propensity methods were applied to mitigate confounding.

Given the study design and potential confounding, these signals should be interpreted cautiously and confirmed in adequately powered, prospective randomized trials. In conclusion, in this single‐center retrospective cohort, both grafts demonstrated favorable outcomes in terms of penile straightening and surgical safety. A higher incidence of postoperative ED was observed with BioA, whereas patients who received SIS reported higher postoperative satisfaction.

## Conflicts of Interest

The authors declare no conflicts of interest.

## Data Availability

The data that support the findings of this study are openly available in PubMed at https://pubmed.ncbi.nlm.nih.gov.
